# A Sweet Connection? Fructose’s Role in Hepatocellular Carcinoma

**DOI:** 10.3390/biom10040496

**Published:** 2020-03-25

**Authors:** Brittany Dewdney, Alexandra Roberts, Liang Qiao, Jacob George, Lionel Hebbard

**Affiliations:** 1 Molecular and Cell Biology, and The Centre for Molecular Therapeutics, Australian Institute of Tropical Health and Medicine, James Cook University, Townsville QLD 4811, Australia; brittany.dewdney@jcu.edu.au (B.D.); alex.roberts@jcu.edu.au (A.R.); 2 Storr Liver Centre, Westmead Institute for Medical Research, Westmead Hospital and University of Sydney, Sydney NSW 2145, Australia; liang.qiao@sydney.edu.au (L.Q.); jacob.george@sydney.edu.au (J.G.)

**Keywords:** fructose, hepatocellular carcinoma, NAFLD, metabolism, NASH

## Abstract

Hepatocellular carcinoma is one of few cancer types that continues to grow in incidence and mortality worldwide. With the alarming increase in diabetes and obesity rates, the higher rates of hepatocellular carcinoma are a result of underlying non-alcoholic fatty liver disease. Many have attributed disease progression to an excess consumption of fructose sugar. Fructose has known toxic effects on the liver, including increased fatty acid production, increased oxidative stress, and insulin resistance. These effects have been linked to non-alcoholic fatty liver (NAFLD) disease and a progression to non-alcoholic steatohepatitis (NASH). While the literature suggests fructose may enhance liver cancer progression, the precise mechanisms in which fructose induces tumor formation remains largely unclear. In this review, we summarize the current understanding of fructose metabolism in liver disease and liver tumor development. Furthermore, we consider the latest knowledge of cancer cell metabolism and speculate on additional mechanisms of fructose metabolism in hepatocellular carcinoma.

## 1. Introduction

Hepatocellular carcinoma (HCC) is the fifth most common cancer and second leading cause of cancer-related deaths worldwide. East Asian countries suffer the greatest disease burden, with more than 40 in 100,000 people affected per year. This is attributed to the regional endemic infections of the hepatitis B virus (HBV) and hepatitis C virus (HCV) [1]. In the US, only 6 in 100,000 people are affected by HCC [2]. However, the incidence rate in the US has tripled since the 1970s and the three-year survival rate is less than 20% [1,2,3]. Additionally, unlike most other cancer types, mortality rates for HCC have significantly increased over the past decade in the US, Australia, and in Northern/Central Europe [4,5]. Therefore, HCC has become the fastest growing cause of cancer-related deaths. 

The major risk factors for HCC are HBV/HCV infections, aflatoxins, alcoholic liver disease, non-alcoholic fatty liver disease (NAFLD), and the inflammatory form of non-alcoholic steatohepatitis (NASH). Most HCC cases present with underlying cirrhosis, often due to viral infection or alcohol abuse. The epidemiology and associated risk factors of viral infection/alcoholic liver disease are well understood. In comparison, the link between NAFLD and the increased HCC incidence is less well described. Males and older individuals (>50 years) with NAFLD are at a greater risk of HCC development [6]. African American and Hispanic individuals with NAFLD may also be at greater risk of HCC. African Americans often present with more advanced stages of HCC and both African Americans and Hispanics with HCC are more resistant to curative therapy [7]. 

Current treatment options for HCC therapy include surgical resection, liver transplantation, radiofrequency ablation (RFA), transarterial chemoembolization (TACE), or medical treatment with sorafenib or regorafenib [8]. Early-stage HCC is usually treated via resection, liver transplantation, or ablation; however, these methods often come with complications [8]. Recurrence of HCC is common post-resection and post-transplantation and occurs between 25% and 75% and 10% and 20% of cases, respectively [9,10,11]. TACE is suitable for intermediate-stage HCC or for multinodular lesions but may cause complications, such as hepatic failure [8,12]. For advanced-stage HCC, only two therapies are available and are used for palliative medical treatment. Sorafenib is a tyrosine kinase inhibitor that targets pathways associated with tumor angiogenesis and proliferation [13]. Moreover, sorafenib is not curative and if effective, may only increase survival by 6–12 months [14,15]. Regorafenib is a similar multikinase inhibitor that is used as a second-line treatment after a failed response to sorafenib [16]. Most diagnoses of HCC occur at the advanced stage where curative treatments are ineffective [14]. Therefore, there is an urgent clinical need for improved treatment options for HCC. 

The development and progression of HCC is highly complex; thus, a deeper understanding of tumor pathogenesis will aid in future therapeutic advances. Recent research has investigated the role of dietary fructose in NAFLD and HCC progression. Excess fructose consumption is well established as a causative factor for developing insulin resistance and fatty liver, thus the rise in NAFLD/NASH cases progressing to HCC in developed countries may be attributed to Westernized diets. However, clinical evidence of fructose association with HCC is limited. This review will address fructose metabolism, the effects on liver and NAFLD pathogenesis, and how the metabolic fate of fructose may affect HCC development. 

## 2. Metabolic Effects of Fructose in NAFLD Development

### 2.1. Glucose Versus Fructose Metabolism

The liver is the main metabolic hub for ingested carbohydrates and absorbs most of the circulating glucose from the blood. Circulating glucose is absorbed by hepatocytes via the glucose transporter type 2 (GLUT2) receptor. In the cytosol, glucokinase (GK) phosphorylates glucose to generate glucose-6-phosphate (G6P), which will either be converted to glycogen for energy storage or proceed through glycolysis for adenosine triphosphate (ATP) production [17,18]. G6P is also a precursor for the pentose phosphate pathway (PPP), which produces NADPH (nicotinamide adenine dinucleotide phosphate) and ribose-5-phosphate, a precursor for nucleotide synthesis [19]. Excess glucose may also be used to generate acetyl-CoA through the citric acid cycle, which utilizes NADPH from the PPP to produce fatty acids [18,19]. Glucose metabolism is tightly regulated by factors such as insulin and glucagon during different nutritional states to maintain metabolic homeostasis. Insulin stimulates insulin receptors IRS1/2 (insulin receptor substrate) to activate a signaling cascade that results in the dephosphorylation of glycogen synthase (GS), thus, increasing glycogen synthesis and regulating hepatic glucose production [19,20]. 

**Figure 1:**
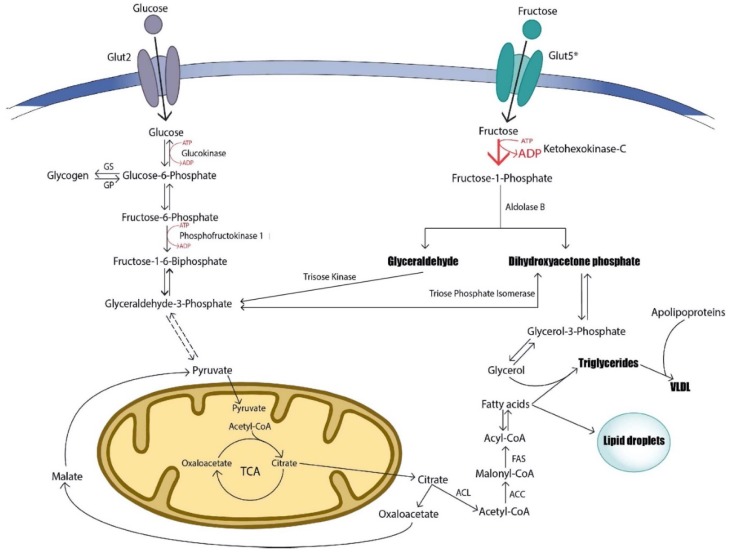
Glucose versus fructose metabolism. Glucose is transported though the Glut2 receptor in the liver and either stored as glycogen or metabolized through glycolysis to produce ATP and pyruvate. Fructose can be transported through Glut2 or Glut5 and is metabolized into trioses through the ATP-dependent enzyme, ketohexokinase-C. Glyceraldehyde and dihydroxyacetone phosphate (DHAP) may be converted to glyceraldehyde-3-phosphate to produce pyruvate or to produce fructose-1,6-biophosphate for gluconeogenesis, or alternatively may directly fuel triglyceride synthesis. The rapid conversion of fructose into trioses may overstimulate the production of triglycerides and VLDL lipoproteins. ACC, acetyl-CoA carboxylase; ACL, ATP citrate lyase; ADP, adenosine diphosphate; ATP, adenosine triphosphate; FAS, fatty acid synthase; Glut2/5, glucose transporter 2/5; GS, glycogen synthase; GP, glycogen phosphorylase; TCA, citric acid cycle; VLDL, very low-density lipoprotein.

Fructose metabolism, however, has another fate within the liver. Fructose is absorbed principally by two GLUT transporter members, GLUT2 and GLUT5 [21]. GLUT5 is primarily expressed in intestinal epithelial cells where fructose is rapidly absorbed and diffused into intestinal capillaries for delivery to the liver via the portal vein, where uptake in hepatocytes occurs through GLUT2 [21,22]. Unlike glucose, fructose metabolism is not regulated by circulating hormones or by negative-feedback loops. Fructose metabolism bypasses the regulated enzymatic reactions involving GK in glycolysis and is rapidly converted to fructose-1-phosphate (F1P) by ketohexokinase (KHK). Two isoforms of KHK are involved in phosphorylating fructose. In the liver, KHK-C is highly active and rapidly phosphorylates fructose into F1P [23,24]. The isoform KHK-A is ubiquitously expressed at low levels and is much less effective at phosphorylating fructose than KHK-C [23,24]. The rapid conversion of fructose in hepatocytes maintains the plasma gradient allowing for continuous fructose uptake in hepatocytes through GLUT2 [25]. F1P is hydrolyzed into dihydroxyacetone phosphate (DHAP) and glyceraldehyde by aldolase B [26]. Both DHAP and glyceraldehyde may be converted by triokinase to glyceraldehyde-3-phosphate (GA3P), which may be used for the glycolytic pathway to produce pyruvate or lactate, or may be used for gluconeogenesis to produce G6P [27]. Additionally, DHAP may be reduced to glycerol-3-phosphate to be a mainstay for de novo lipogenesis (DNL), allowing the synthesis of triglycerides (TG) and lipoproteins [27]. The basic metabolic differences in glucose and fructose metabolism have been well summarized previously (Figure 1) [28,29].

### 2.2. Fructose and Insulin Resistance

Fructose has been shown to increase the activity of proteins involved in lipogenesis, gluconeogenesis, and glycolysis. Transcription factors carbohydrate-responsive element-binding protein (ChREBP) and sterol regulatory element binding protein-1 (SREBP-1) are potent inducers of lipogenesis by activating genes such as fatty acid synthase (FAS) and acetyl coA carboxylase (ACC) [25], and have been demonstrated to have increased activity in response to fructose feeding [30,31,32,33]. Additionally, fructose increases the expression of the gluconeogenic enzymes glucose-6-phosphatase (G6Pase) and fructose 1,6-biphosphatase 1 (FBP1), the glycolytic enzymes phosphofructokinase 1 (PFK1) and pyruvate kinase (PK), and increases glycogen and hepatic glucose production [32,33]. Therefore, it can be concluded that fructose increases the expression of genes that allow the conversion of fructose-derived ketoses into glucose or to pyruvate for endogenous glucose and TG synthesis, while inhibiting exogenous glucose uptake. Research also suggests that the regulation of these genes is mediated by the fructose-induced activity of ChREBP and that this activity dominates the suppressive effects of insulin on gluconeogenesis [25,33]. Therefore, the gluconeogenic and lipogenic action of fructose, irrespective of insulin signaling, may contribute to hepatic insulin resistance. Furthermore, some studies have demonstrated that a high fructose diet can reduce the phosphorylation of IRS1 and expression of IRS2 [34,35,36]. However, more research is required to verify potential direct or indirect effects of fructose on insulin receptor activity. 

### 2.3. Fructose and Oxidative Stress

**Figure 2:**
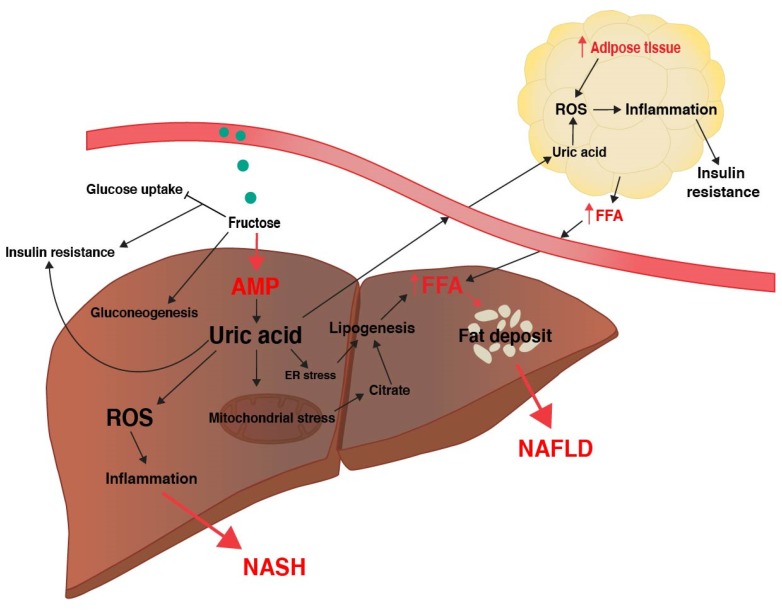
Fructose and liver disease pathogenesis. Fructose sugar is rapidly metabolized by the liver and causes a depletion of ATP, and results in uric acid accumulation. Uric acid causes metabolic stress on liver organelles, such as the mitochondria, leading to increased citrate production and lipogenesis. Uric acid may also circulate to the adipose tissue and cause adipocyte dysfunction and inflammation, increasing circulating free fatty acids that may in turn contribute to fat deposition within the liver. Uric acid also increases reactive oxygen species, leading to liver inflammation. Fructose metabolism may additionally induce insulin resistance through inhibiting glucose uptake and stimulating gluconeogenesis. AMP, adenosine monophosphate; ER, endoplasmic reticulum; FFA, free fatty acids; NAFLD, non-alcoholic fatty liver disease; NASH, non-alcoholic steatohepatitis; ROS, reactive oxygen species.

Fructose conversion to F1P occurs rapidly and is ATP-dependent, thus the ingestion of fructose results in ATP and intracellular phosphate depletion and increased intracellular adenosine monophosphate (AMP) [37,38]. Consequently, the activation of AMP deaminase occurs to stimulate nucleotide degradation, converting AMP to inosine monophosphate (IMP) in an attempt to restore phosphate and ATP levels, and results in the accumulation of uric acid as a by-product [39]. Intracellular uric acid causes mitochondrial oxidative stress, production of reactive oxygen species (ROS), and increases hepatic fat accumulation [40]. Increased mitochondrial oxidative stress inhibits enzymes involved in citrate metabolism [40], and accordingly mitochondrial citrate accumulates and is released into the hepatocyte cytosol where it activates ATP-citrate lyase and FAS, and increases hepatic DNL [40]. Additionally, uric acid causes endoplasmic reticulum stress, which also stimulates the expression of lipogenic enzymes such as ACC and FAS through increased SREPB-1 signaling [41]. Increased TG synthesis in the liver overloads the ER with lipids for very low-density lipoprotein (VLDL) synthesis, further promoting ER stress [42]. Hepatocyte release of uric acid into the circulation may also cause adipocyte uptake of uric acid and induce the activation of NADPH oxidase to generate ROS and oxidative stress in adipocytes [43]. The effects of uric acid on oxidative stress may occur independently of fructose and excess caloric intake [40,41,43]. In addition, circulating uric acid may promote insulin resistance in liver, fat, and muscle tissue by increasing serine phosphorylation of IRS1 and inhibiting insulin responses [44]. Thus, through rapid deletion of ATP, and the subsequent generation of uric acid, both hepatocytes and adipocytes are exposed to high levels or ROS that may contribute to DNL, insulin resistance, and NAFLD (Figure 2).

### 2.4. Fructose, Inflammation, and NASH

The potential mechanisms of fatty liver development discussed above may also contribute to hepatic inflammation and consequently a progression to NASH. Fructose-induced accumulation of uric acid generates ROS both in hepatocytes and adipocytes (Figure 2), which induces a pro-inflammatory signaling cascade through the release of cytokines [45]. Xanthine oxidase (XO) catalyzes the reaction converting xanthine into uric acid, thus fructose-induced accumulation of uric acid follows an increase in XO activity [46]. XO acts as an electron donor for oxygen, thus generating ROS and inducing oxidative stress in liver [46,47]. Furthermore, uric acid directly activates the inflammasome NLRP3 (NLR family pyrin domain containing 3), which is responsible for the maturation of IL-1/18 (interleukin 1/18) [46,48,49,50]. The activation of NLRP3 is a significant step in the progression of NAFLD to fibrosis and NASH as it activates immune system modulators responsible for stimulating fibrosis and inflammation [51,52,53]. Continuous stimulation of fibrotic and chronic inflammation damages hepatocytes and may lead to cirrhosis and HCC. 

Furthermore, the cascade of inflammatory pathways induced by excess fructose consumption may also contribute to the activation of stress hormones involved in the hypothalamus–pituitary–adrenal (HPA) axis. In response to the fructose-induced inflammation in hepatocytes and adipocytes, the HPA axis is activated to synthesize immunosuppressors, such as glucocorticoids [54,55]. This in turn increases the activity of 11-β-hydroxysteroid dehydrogenase type 1 (11β-HSD-1), an important enzyme that activates glucocorticoids, such as cortisol, and is largely involved in metabolic-related diseases [56]. Increased bioactivity of cortisol may stimulate lipogenesis and lead to visceral fat deposition and hepatic fat deposition [54,55,57]. Therefore, a fructose-induced disruption of the regulation of stress hormones could in part contribute to NAFLD and NASH.

### 2.5. Fructose and the Microbiome

It has been well established through several animal and human studies that small intestine microbiome dysbiosis is associated with NAFLD/NASH development, as demonstrated by increased endotoxin levels and intestinal permeability [58]. Although the precise mechanisms have yet to be elucidated, studies have shown that fructose consumption can elevate endotoxin translocation from the gut to the liver [59,60,61,62,63]. This is likely due to the fact that fructose feeding is associated with a loss of epithelial cell tight junctions, such as occludin and claudin-1, allowing endotoxins to enter the liver and stimulate inflammatory reactions that could in turn contribute to NASH [64,65,66]. Furthermore, the abundance and diversity of intestinal flora may be altered due to excess fructose exposure. Studies have described the effects of both high fructose and high fat diets on the relative ratio of Firmicutes/Bacteroidetes, where in diets high in fructose or states of metabolic disorder, there is a decrease in the relative abundance of Bacteriodes [59,66,67,68], which usually dominate the intestinal tract in a healthy individual [69]. Of note, research has shown that the addition of some probiotics, such as strains of Lactobacillus, have protective effects and may restore the gut function after high fructose exposure by increasing the levels of beneficial bacteria and restoring the intestinal barrier [64,70,71]. Nevertheless, due to the effects of fructose, sugar and Westernized diets have become an emerging topic in health and metabolic-related diseases. This is still a complex field of study that requires further investigation, especially as it pertains to the development of chronic liver conditions, such as NASH. Moreover, due to the high dietary levels of fructose in these studies, whether the findings are relevant to the human condition is questionable. 

## 3. Clinical Observations of Fructose Metabolism in NAFLD

### 3.1. Epidemiological Association of Fructose and NAFLD

Current epidemiological studies show that the percentage of obese patients with NAFLD ranges from 30% to 75%, whereas NAFLD in non-obese patients shows a surprisingly high range of 5%–30%. The prevalence of NAFLD in the population varies substantially not only due to ethnical and environmental differences, but also due to the poor accuracy of ultrasounds while they are the most used method of diagnosing liver steatosis. Lean NAFLD cases have been characterized by visceral obesity and low or even absent hepatic insulin resistance (IR) [72]. The development of NAFLD in lean subjects may be attributed to adipose IR and visceral obesity even though hepatic IR remains normal, causing an increased release of free fatty acids (FFA) from visceral fat, and the deposition of fats within the liver due to drainage into the portal circulation [72,73]. 

While the precise mechanisms in which NAFLD develops in non-obese patients has yet to be fully elucidated, dietary factors are likely to be a major contributor. One study has shown non-obese NAFLD patients to consume more than double the amount of sugar per day than the matched healthy controls, where nearly half of this sugar came from soft drinks [74]. Additionally, this study shows the overall caloric intake and macromolecule diet composition to be similar in both groups, demonstrating that excess consumption of high fructose soft drinks was the only independent variable associated with fatty liver diagnosis [74]. Another study shows similar results in obese patients with and without metabolic syndrome, where the excess sugar consumption was significantly higher in both groups compared to the healthy controls [75]. Excess soft drink consumption was the strongest predictor of fatty liver in these patient groups, irrespective of the diagnosis of metabolic syndrome [75]. Moreover, additional reports have predicted that lean individuals that consume diets higher in fructose are believed to be more likely to develop fatty liver, even when caloric intake is within normal range [72,73,74]. 

### 3.2. Fructose Metabolism in Humans is Variable

Some studies have shown small amounts of fructose in the presence of glucose can improve glucose uptake and decrease hyperglycemia in normal and diabetic subjects, particularly in states of exercise [76,77,78,79]. However, excess consumption of fructose may significantly reduce circulating insulin, and possibly impair hepatic glucose metabolism [80,81,82]. Several short-term studies have shown that fructose feeding, either in weight maintaining diets or hypercaloric diets, in healthy subjects may increase intrahepatocellular lipids, DNL, and blood triglyceride levels [83,84,85,86]. Additionally, some of these studies show that the consumption of fatty acids and coffee may attenuate the effects of very low-density lipoprotein (VLDL) production induced by fructose consumption [85,86,87]. 

Other short-term investigations in obese children have shown that restricting fructose consumption reduces liver fat, DNL, and improves insulin sensitivity [88,89]. In contrast, studies involving fructose consumption in overweight/obese patients show that long-term or short-term consumption of excess fructose or glucose made no difference in parameters of metabolic-related conditions, such as inflammation, liver triglycerides levels, serum triglycerides, or insulin levels [90,91,92]. Furthermore, one other long term study shows contradicting results, and it demonstrated that long term fructose consumption could induce lipogenic and insulin resistance effects in obese patients [93]. Together, it is clear that there are contradicting pieces of evidence regarding the metabolic effects of fructose in healthy versus overweight subjects.

However, considering that the majority of this clinical data was obtained over short time periods of 7–14 days, and the number of longer-term studies is limited, it remains unclear whether fructose alone is solely responsible for changes in fat metabolism and insulin levels in patients. Furthermore, there is a large range in the amount of fructose used in clinical studies varying from 20% to 35% of total energy requirements, either as weight-maintaining diets or hypercaloric diets [83,84,85,90]. Interestingly, an earlier evaluation of the estimated fructose consumption in the United States showed that the average consumption of total fructose only represented between 8% and 12% of total energy. This equated to approximately 1 gram/kilogram of body mass, whereas most clinical studies of fructose consumption use diets containing approximately 3.5 grams/kilogram of body mass. 

In healthy individuals, fructose circulates in the bloodstream at a significantly lower amount of just 5.5 μM in the fasted state, as compared to 4.5 mM for circulating glucose [94]. However, on ingestion, fructose levels rise many-fold more than those of glucose, increasing to nearly 300 uM and remain elevated in the bloodstream for one to two hours longer than glucose [94]. Therefore, it is unclear if the clinical studies using high fructose diets truly reflect what is normally consumed in the Westernized diet, or if the extreme amounts of fructose used in these studies are the major reason for causing lipogenic effects. 

Several meta-analyses display this controversy by indicating the large number of heterogenic studies that contribute to differences in fructose metabolism in healthy versus overweight patients. By example, a meta-analysis revealed that isocaloric diets supplemented with fructose over glucose showed no increase in postprandial triglycerides [95]. However, when the studies only investigating overweight subjects were considered, fructose significantly increased triglycerides compared to the supplementation with glucose [95]. In studies with hypercaloric diets, fructose supplementation significantly increased postprandial triglycerides in both normal and overweight subjects [95]. 

In contrast to the above, another meta-analysis identified fructose feeding to be positively associated with fasting blood sugar levels and increased triglycerides [96]. However, when the studies that significantly contributed to study heterogeneity were eliminated, the association of fructose with the symptoms of the metabolic syndrome were not statistically significant [96]. Another meta-analysis showed fructose consumption to increase hepatic insulin resistance in isocaloric and hypercaloric healthy non-diabetic subjects, but had no effect on extrahepatic insulin resistance [97]. It may therefore be concluded that the effects of fructose on metabolism vary quite substantially, and likely contribute to liver disease alongside other factors, such as body mass index (BMI), metabolic disorders, and other dietary factors.

## 4. A challenging Theory—Does Fructose Contribute to HCC Development?

### 4.1. Association of Fructose Consumption and Cancer Risk

The progression of NASH to HCC is likely to be multifactorial, including factors such as oxidative stress and chronic inflammation leading to damaged hepatocytes and the stimulation of fibrosis, lipotoxicity, and genetic mutations. NASH is a major risk factor for HCC, with 4%–22% of HCC cases relating to underlying NAFLD and NASH [98]. However, it remains unclear how fructose contributes to HCC development. Studies have investigated the association of sugar consumption with general cancer risk, but with varying associations based on the sugar and cancer type in question. Reports have shown a positive association with sugar consumption, either as glucose, fructose, or sugar-sweetened beverages, and pancreatic cancer [99,100,101,102,103]. However, some larger studies showed no association with sugar consumption and an increased risk of cancer development [104,105,106]. Furthermore, two NIH-American Association of Retired Persons (AARP) studies have shown that additive fructose has limited association with overall cancer risk of the major cancer types or cancer-related mortality [107,108]. Specifically, fructose intake was weakly associated with a decreased risk for all cancers in men, and a decreased risk of liver and ovarian cancer in women [107]. For both sexes, fructose consumption only associated with an increased risk of small intestine cancer [107]. However, a more recent study showed no association with fructose intake and risk of cancer-related mortality in men or women [108]. 

Regarding HCC, there are limited studies that have investigated the relationship of fructose consumption and HCC risk. Some studies have shown a positive association between glucose and the risk of HCC, where a sample of 147 and 250 respective HBV/HCV HCC patients with a high glycemic load had an increased overall risk (OR) of HCC compared to control (OR 3.25 and OR 1.95, respectively) [109,110]. Another study showed that in 191 HCC patients there was a positive association with high total sugar intake and HCC (Hazard Ratio 1.88) [111]. However, the link between fructose consumption and HCC remains poorly investigated, and the limited data that we present below concerning the topic is controversial. Therefore, there is a need for future studies to determine the associated risks of HCC with the consumption of diets high in fructose sugar.

### 4.2. Traditional Animal Models Suggest High Fructose Promotes HCC Development

Some animal studies have demonstrated that fructose containing diets increase the incidence of liver tumor growth. In one study, using the carcinogen diethylnitrosamine (DEN), mouse HCC was evaluated using diets that included (i) normal chow, (ii) high sucrose/high lard fat, (iii) high sucrose/high coconut oil fat, (iv) high sucrose/fructose and low fat, and (v) and high lard/low sugar. Healy and colleagues demonstrated a similar tumor incidence among male mice fed the above diets [112]. However, the tumor burden was significantly increased in the high sucrose/high lard fat and the high sucrose/fructose and low fat groups, compared to the high lard/low sugar and normal chow diets. Histology demonstrated the high sucrose/high lard fat liver tumors to have 30% HCC morphology compared to the high sucrose/fructose and low fat, which had only 10% HCC foci [112]. Thus, in this model the combination of a high carbohydrate and high fat diet contributed to HCC development rather than high fructose alone. 

It is known that males are at a greater risk of attaining HCC. Therefore, using the above models, the same investigators observed that female mice had increased tumor incidence when fed the high sucrose/high lard fat, and high sucrose/fructose and low fat diets, compared to the high lard/low sugar and normal diets [113]. Interestingly, the tumor burden was highest in high sucrose/high lard fat diet and the high sucrose/fructose and low fat diet. The high sucrose/high coconut fat diet had the lowest tumor burden [113]. Similar to the male mice, in female mice the high sucrose and high fat was the greatest contributor to tumor development, although high sucrose/fructose and low fat also increased tumor growth compared to normal chow [113]. Taken together, sex appears to play a significant role in the metabolic contributions of fructose to HCC development. 

Another study evaluating DEN-treated rats on an eight-week diet of either high-fat, high-fructose, or normal chow reported somewhat controversial results. In this study, pre-cancerous cells were more prominent in the high-fructose compared to the high-fat diet [114]. Liver oil red O staining revealed the high-fat diet to have significantly promoted more hepatic fat deposits compared to the high-fructose diet, even though the liver weights remained similar between diet groups [114]. The short duration of diet feeding and differences in diet composition likely caused these differences. Recently, a new murine model of NASH-HCC has been developed that utilizes a combination of a diet high in fat and fructose with chemical treatment of carbon tetrachloride (CCl_4_) to induce fatty liver, fibrosis, and HCC [115]. Collectively, these results suggest a combination of high fat and high fructose to be a promoter of HCC development.

### 4.3. Genetic Changes Associated with HCC May Reduce Fructose Metabolism 

While there is limiting clinical evidence regarding the genetic changes associated with fructose exposure in HCC, there are several studies that imply reduced fructose metabolism in HCC. A recent study has demonstrated this in vitro, evident by a metabolic switch from KHK-C expression to predominant expression of KHK-A in HCC [116], which has a low affinity and metabolic flux for fructose. Additionally, the authors showed that KHK-A activates downstream enzymes involved in activating the PPP, thus contributing to HCC development [116]. While this study confirmed that liver cancer cells had significantly reduced fructose metabolism compared to normal hepatocytes, the effects on cell proliferation were not evaluated. Similarly, another study showed that human HCC tumors had reduced aldolase B expression compared to the paired non-cancerous tissue, and this was associated with reduced survival [117,118]. Aldolase B overexpression can also decrease tumor cell migration and inhibit HCC-related metastasis [118]. Studies have also investigated the role of FBP1 as a prognostic marker of HCC. FBP1 is a gluconeogenic enzyme that is upregulated in response to fructose exposure [33]. Clinical research has shown that low FBP1 expression is associated with poorer survival and more aggressive HCC growth [119,120,121,122,123,124]. Low FBP1 expression enhances glycolysis in HCC [122,124], while increasing FBP1 reduced GLUT1 expression, glucose uptake, lactate production, and cell growth [120,121,123]. KHK-C, aldolase B, and FBP1 all increase after fructose consumption and are required for the breakdown of glycolytic products. Together, these data suggest that fructose is poorly metabolized by HCC, or that it inhibits tumor cell growth in a glycolysis-dependent cell. 

### 4.4. Fructose May Promote Metabolic Adaptations of the Traditional Warburg Effect

Tumor progression is dependent on acquired characteristics that allow the growth and development of cancerous cells, commonly known as the hallmarks of cancer [125]. The hallmark that describes the ability of cancer cells to reprogram energy metabolism is of interest to this review, in particular glucose metabolism to favor the glycolytic pathway, otherwise known as the “Warburg effect” [126]. Otto Warburg first described the altered metabolism of cancer cells in 1930, where in aerobic conditions cancer cells favor generating ATP and lactate from glycolysis. Later studies confirmed that this caused the build-up of glycolytic metabolites that feed into the PPP to result in the generation of more nucleotides, NADPH, and lipids for multiplying tumor cells [127,128,129]. Mechanistically, the availability of growth factors and glucose will push cancer cells towards glycolysis for the purpose of generating energy and biomolecules. Growth signals, such as epidermal growth factor receptor (EGFR), activate the PI3K/Akt signaling pathway and consequently increase glucose transporters (GLUT1) expression and the activity of PFK2 [130]. PFK2 phosphorylates fructose-6-phosphate (F6P) to generate fructose-2,6-biphosphate (F-2,6-BP), which promotes glycolysis by activating PFK1 and in turn inhibits gluconeogenesis through inhibiting FBP1 [130,131]. Hence, increased F-2,6-BP stimulates cancer cell glycolysis to fuel cell growth. This state is also induced by high levels of glucose and insulin, which may further induce F-2,6-BP production [132].

However, recently our understanding of cancer metabolism has shifted to a more dynamic view. This is termed the “reverse Warburg effect”, whereby anabolic cancer cells metabolically adapt to the tumor microenvironment and promote aerobic glycolysis in the neighboring stromal cells. The stromal cells produce lactate, fatty acids, amino acids, glutamine, and ketone bodies that are in turn used by the cancer cells to support growth [131,133,134,135]. In response to metabolic stress, cancer cells may shift away from glycolysis and primarily utilize the PPP and mitochondrial oxidative phosphorylation as the primary means of obtaining energy, producing nucleotide precursors, and generating antioxidants to manage ROS. This switch is in part driven by the p53-induced TP53-induced Glycolysis and Apoptosis Regulator (TIGAR), which has a similar function to that of FBP1. TIGAR inhibits glycolysis by converting F-2,6-BP into F6P, which increases the flux of carbons into the PPP to generate NADPH and nucleotides [136]. Importantly, an increased TIGAR expression is associated with cancer cell growth and tumor progression [131,137,138,139]. TIGAR expression can also induce monocarboxylate transporter (MCT) expression, allowing cancer cells to use catabolites from the neighboring metabolically reprogrammed stromal cells. In this manner, the stromal cells increase glycolysis to produce lactate, which is supplied to the growing cancer cells [135,140]. Cancer cells then use the citric acid cycle to utilize uptake of lactate and other catabolic metabolites for the production of energy and antioxidants [131,140,141]. Such compounds also include glutamine, which is metabolized to glutamate to fuel the citric acid cycle through α-ketoglutarate [142,143], and the generation of glutathione (GSH), a potent antioxidant that detoxifies ROS and carcinogens that accumulate in active and metabolically stressed cancer cells [144]. Significantly, increased GSH associates with elevated HCC proliferation and growth [145,146] and contributes to chemo-resistance [147,148].

**Figure 3:**
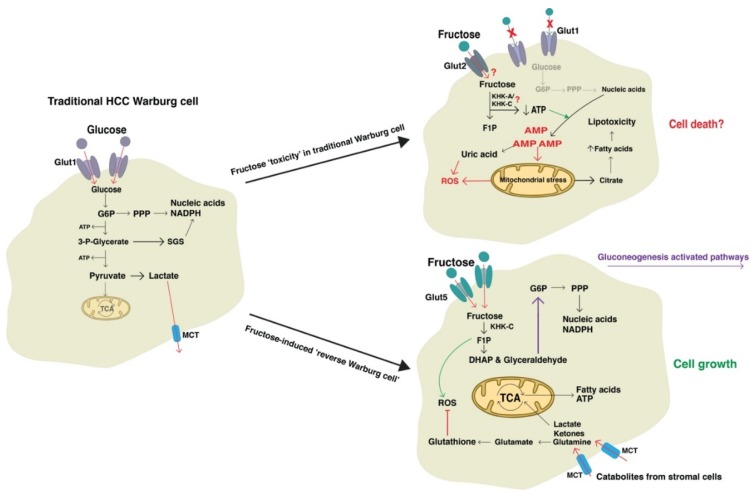
Potential consequences of fructose metabolism in hepatocellular carcinoma (HCC)**.** A traditional HCC cancer cell may exhibit the Warburg effect where glycolysis is favored to produce ATP and glycolytic products for nucleotide synthesis. If this cancer cell is exposed to fructose as the only source of fuel, the toxic effects of fructose may be deleterious to the cell by depleting energy and increasing ROS, leading to cell death. However, in cancer cells exposed to high concentrations of fructose, the cancer cell may metabolically adapt to sustain growth through activation of the MCT and gluconeogenic enzymes. In this case the cell switches to the ‘reverse Warburg effect’ to utilize fructose by-products to fuel nucleotide synthesis and to uptake catabolites from neighbouring cells for production of energy and antioxidants. ATP, adenosine triphosphate; AMP, adenosine monophosphate; DHAP, dihydroxyacetone phosphate; F1P. P, fructose-1-phosphate; G6P, glucose-6-phosphate; Glut1, glucose transporter 1; Glut2, glucose transporter 2; Glut5, glucose transporter 5; KHK-A, ketohexokinase-A; KHK-C, ketohexokinase-C; MCT, monocarboxylate transporters; PPP, pentose phosphate pathway; ROS, reactive oxygen species; SGS, serine to glycine synthesis pathway; TCA, tricarboxylic acid cycle (citric acid cycle).

According to these authors, integrating these observations with the current knowledge on fructose metabolism, results in a conflicting model of cancer development. The metabolic fate of fructose causes rapid depletion of ATP levels, increased cellular ROS, and increased gluconeogenesis. It could be expected that in HCC cancer cells that display the Warburg effect, a high fructose diet would not supply cancer cells with the appropriate fuel. The rapid conversion to F1P would be deleterious to proliferating cells due to ATP depletion, nucleotide degradation, and the accumulation of uric acid and ROS. As the Warburg effect encourages glycolysis, we would not expect much fructose to enter the PPP and promote nucleotide synthesis. It would be expected that the overload of ROS in association with depleted energy and nucleotides would lead to apoptosis. Additionally, as discussed in previous sections, the enzymes involved in fructose metabolism are thought to act as HCC tumor suppressors. Thus, it is conceivable that while fructose may contribute to NAFLD pathogenesis, HCC fed by fructose may lead to growth inhibition (Figure 3). Although, in the clinical setting this is not relevant as fructose is not the sole energy source for the human diet. Nevertheless, knowledge of the biochemical consequences of fructose metabolism in cancer cells is novel and warrants further investigation. It is possible that such studies could reveal new ways to target cancer cell growth. 

Taking into account the current evidence, we suggest that fructose consumption more likely drives cancer cells into a metabolically stressed state, inducing the reverse Warburg effect to maintain cell growth and control the increase in cellular ROS. This is exemplified by the fact that fructose consumption has been shown to increase expression of FBP1 as well as increase enzymatic activity of glucose-6-phosphate dehydrogenase (G6PD), a key enzyme in the PPP [33]. Furthermore, fructose induces expression of ChREBP, which is a known activator of TIGAR [33,149]. Thus, it could be concluded that cancer cells adapt to fructose-induced metabolic stress by increasing gluconeogenesis and the PPP, increasing glutamine uptake to reduce ROS, and utilizing catabolites from the tumor microenvironment (Figure 3). While the reverse Warburg effect supports this theory, more research is required to confirm the action of fructose on promoting or inhibiting HCC metabolism. Such studies also need to consider the relevancy of the human diet where glucose and fat consumption is the norm.

## 5. Conclusions

Fructose metabolism has been implicated in the development and progression of NAFLD and NASH. Fructose metabolism in the liver bypasses the regulatory enzymes of glycolysis and is believed to result in increased lipogenesis, oxidative stress, and inflammation. Some studies have utilized high-fructose diets to study HCC development; however, it remains unclear how these models may translate to clinical NASH-HCC. Our current knowledge on traditional cancer metabolism and the genetic changes associated with HCC suggest that fructose as a fuel source may restrict tumor growth. However, while fructose may induce metabolic stress in cancer cells, it is likely that metabolic reprogramming will occur to allow continued tumor growth. A better understanding of the role of fructose in NAFLD-HCC development and the metabolic fates of fructose in cancer cells will allow for more specific drug therapies to be developed. Targeted treatment options are required to more effectively treat the increasing number of NAFLD-HCC cases arising in developed countries.
